# Prognostic values of modified NUTRIC score to assess outcomes in critically ill patients admitted to the intensive care units: prospective observational study

**DOI:** 10.1186/s12871-023-02086-0

**Published:** 2023-04-20

**Authors:** Ata Mahmoodpoor, Sarvin Sanaie, Tohid Sarfaraz, Kamran Shadvar, Vahid Fattahi, Hadi Hamishekar, Amir Vahedian-Azimi, Abbas Samim, Farshid Rahimi-Bashar

**Affiliations:** 1 Neurosciences Research Center, Aging Research Institute, Tabriz University of Medical Sciences, Tabriz, Iran; 2 Department of Anesthesiology, Faculty of Medicine, Tabriz University of Medical Sciences, Tabriz, Iran; 3 Department of Anesthesiology, Faculty of Medicine, Tabriz Islamic Azad Uniersity, Tabriz, Iran; 4 Department of Clinical Pharmacy, Faculty of Pharmacy, Tabriz University of Medical Sciences, Tabriz, Iran; 5 Trauma research center, Nursing Faculty, Baqiyatallah University of Medical Sciences, Tehran, Iran; 6 Chemical Injuries Research Center, Systems Biology and Poisonings Institute, Baqiyatallah University of Medical Sciences, Tehran, Iran; 7 Evidence Based Research center, Tabriz University of Medical Sciences, Tabriz, Iran; 8 Anesthesia and Critical Care Department, Hamadan University of Medical Sciences, Ayatolah Motahari BLVD Resalat Square, Hamadan, 6514845411 Iran

**Keywords:** Intensive care unit, mNUTRIC score, Mortality, Nutritional adequacy

## Abstract

**Purpose:**

Modified Nutrition Risk in the Critically Ill (NUTRIC) score (mNUTRIC score) have been validated as screening tool for quantifying risk of adverse outcome critically ill patients admitted to the intensive care units (ICUs). The aim of this study was to evaluate the prognostic value of mNUTRIC score to assess outcomes in this population.

**Materials and methods:**

This prospective, observational study was conducted on adult patients admitted to the general ICUs of two university affiliated hospital in northwest of Iran. The association between the mNUTRIC score and outcomes was assessed using the univariate and multivariate binary logistic regression. The performance of mNUTRIC score to predict outcomes was assessed using the receiver operating characteristic (ROC)-curve.

**Results:**

In total 445 ICU patients were enrolled. Based on mNUTRIC score, 62 (13.9%) and 383 (86.1%) individuals were identified at high and low nutritional risk, respectively. The area under the curve (AUC) for predicting ICU mortality, using vasopressor, duration of vasopressor, and mechanical ventilation (MV) duration were (AUC: 0.973, 95% CI: 0.954–0.986, P < 0.001), (AUC: 0.807, 95% CI: 0.767–0.843, P < 0.001), (AUC: 0.726, 95% CI: 0.680–0.769, P < 0.001) and (AUC: 0.710, 95% CI: 0.666–0.752, P < 0.001), respectively.

**Conclusions:**

An excellent and good predictive performance of the mNUTRIC score was found regarding ICU mortality and using vasopressor, respectively. However, this predictive was fair for MV and vasopressor duration and poor for ICU and hospital length of stay.

## Introduction

Nutritional support is an important part of the care of critically ill patients [1]. Nutrition Risk in critically ill patients is associated with poor outcomes, including high nosocomial infections rates, low wound healing rates, high mortality rates, and increased hospital-related cost [2, 3]. The nutritional status of patients admitted to the intensive care unit (ICU) is impaired by both chronic and acute starvation, with many catabolic processes such as faster lean body mass loss and single or multiple organ failure [4, 5]. It is important to identify patients at risk by assessing nutritional status within 48 h of hospital admission. Various scoring systems, criteria, and tools are used to assess nutritional risk, including physical examination, dietary intake, functional assessment, severity of illness and anthropometric data [6–8]. However, most of these nutritional assessment tools and criteria do not consider inflammatory processes and hypermetabolic status/muscle wastage in ICU patients [9–11].

The Nutrition Risk in Critically Ill (NUTRIC) score was introduced by Heyland et al. [12], to identify critically ill patients who will benefit from aggressive protein-energy therapy during their stay in the ICU. This will improve mortality and mechanical ventilator (MV) duration [12, 13]. During the development of the NUTRIC-score, the specific baseline characteristics evaluated to stratify the effects according to the impact baseline risk of nutritional interventions on ICU patients [14–16]. NUTRIC-score includes age, number of comorbidities, days from admission to ICU admission, disease severity based on Acute Physiology and Chronic Health Evaluation II (APACHE-II) and Sequential Organ Failure Assessment (SOFA) scores on ICU admission, and Interleukin-6 level (IL-6) as an optional variable to assess nutritional risk and associated outcomes such as mortality, MV duration, duration of vasopressor, ICU and hospital length of stay (LOS). The measurement of IL-6 is not routinely available in most ICUs. Therefore, in settings where IL-6 is not available, this may be removed from the NUTRIC-score. This adjusted score is called the modified NUTRIC score (mNUTRIC), which is a combination of seven clinical and laboratory parameters: age, BMI, APACHE II score, serum albumin, serum creatinine, serum sodium, and serum glucose. The mNUTRIC score has been shown to be a reliable and valid predictor of mortality in critically ill patients, and is associated with a significant increase in mortality when the score is greater than or equal to 4. The mNUTRIC score is used to identify patients at high risk for poor outcomes, and to guide decisions about nutritional support. In addition, it is recommended that clinicians use the mNUTRIC score to assess nutritional risk and guide decisions about nutritional support in critically ill patients [17–19].

Identifying critically ill patients at high nutritional risk is important for reducing morbidity and mortality, so the need for easy-to-implement, inexpensive, and highly effective scores is undeniable. NUTRIC score appear to be effective but the inclusion of costly IL6 measurements makes them unattractive for widespread implementation. Therefore, mNUTRIC appear to be the most promising tool for nutritional risk assessment and requires further validation [13]. The aim of this study was to evaluate the prognostic value of the mNUTRIC score for evaluating outcomes in critically ill patients admitted to the ICU.

## Methods

### Study Design and setting

This prospective observational study was conducted to evaluate the prognostic value of mNUTRIC score for assessing outcomes in critically ill patients admitted to the general ICUs in two university-affiliated hospitals (Shohada and Imam Reza Hospitals) in northwest of Iran between 21 and 2019 and 19 March 2021. The protocol study was reviewed and approved by Research Ethics Committees of Islamic Azad University-Tabriz Branch (IR.TBZMED.REC.1398.021), in accordance with the Declaration of Helsinki of the World Medical Association [20]. Written informed consent was obtained from the patients or from their legally accepted representatives. In addition, the study was conducted and reported in accordance with the recommendations of the Strengthening the Reporting of Observational Studies in Epidemiology (STROBE) statement [21].

### Study population

All adult patients (at least 18 years of age) admitted to the general ICUs for more than 48 h were included in the study and followed up to evaluate the study hypothesis until discharge from the ICU or death in the ICU. Patients were excluded if they were: (a) diagnosed as brain dead at admission or during ICU stay, (b) patients with COVID-19, (c) those transferred to another ICU or hospital, (d) those readmitted to the ICU, (e) patients who discharged or died within 24 h after ICU admission and (f) if data on m-NUTRIC score was incomplete.

### Data collection

Demographic characteristics (age and sex), baseline clinical data such as comorbidities, type of comorbidities, reason for ICU admission, place of admission (surgery room, emergency, department and elective), severity of illness (based on APACHE II and SOFA score) and blood culture (negative vs. positive) were obtained 24 h after admission. In addition, nutrition assessment included the nutrition administration route (parenteral, enteral or both), amount of energy (kcal/kg), amount of protein intake (gr/kg), gastric residual volume (GRV > 200 cc), and dysglycemia (according to fasting glucose levels, postprandial glucose levels, hemoglobin A1C levels, and glucose tolerance tests) were collected for each patient. GRV is measured just before the next enteral feeding by suctioning of enteral tube and measurement of the amount of withdrawn volume. We measured GRV before every feeding which was 7 times per day. Nutritional adequacy was defined as the difference between energy intake and energy required. The amount of energy requirement as 25 kcal/kg was calculated by weighing the patients. Standard formula for enteral nutrition was used for patients (1 kcal/ml), so the energy intake by the amount of enteral nutrition which is received by patients was calculated. By subtracting this amount by the required energy, the nutritional adequacy was calculated.

. The energy and macronutrient requirements were calculated considering the American Society for Parenteral and Enteral Nutrition (A.S.P.E.N.) guideline. Based on the guideline the amount of required energy in obese patients is based on ideal body weight and in normal and underweighted patients is based on real body weight [22, 23].

### Calculation of mNUTRIC score

The mNUTRIC score was calculated based on age, APACHE II, SOFA, number of comorbidities, number of days between hospital admission and ICU admission. The mNUTRIC score was calculated within a range of 0 and 9 score. A total score of 4 and below (≤ 4) displays low nutrition risk, while above of 4 score (> 4) shows high nutrition risk [12, 13]. The calculation of mNUTRIC score was performed in the first 48 of ICU admission.

### Outcomes

The primary outcome was ICU mortality and ICU length of stay (LOS), hospital LOS, MV and MV duration, using vasopressor and duration of using vasopressor were the secondary outcomes.

### Statistical analysis

All studied variables were tested for normal distribution with Kolmogorov-Smirnov test. Continues variables are reported as mean ± standard deviation (SD) or/ and median (interquartile range: IQR), and categorical variables are presented in frequencies and percentages. The nutrition characteristics and also outcomes including use of MV, MV duration, use of vasopressor, duration of vasopressor, ICU mortality, hospital and ICU LOS, in patients with low (≤ 4) and high (> 4) mNUTRIC scores were compared using the Chi-Square or Fisher’s Exact tests for binary variables and the t-test for normally distributed continuous variables or two-tailed Mann–Whitney test as nonparametric for not normally distributed continuous variables. Univariate and multivariate binary logistic regression model analysis were used to evaluate associations of various baseline parameters and mNUTRIC score to predict outcomes and the results were expressed as odds ratio (OR) with 95% confidence interval (CI). The variables are classified based on the median. For example, patients aged < 64 and > 64 years, ICU stay > 6 days, duration of MV > 6 days or not. In the univariate model, each variable was entered in the model. However, in the multivariate model, all variables were entered in the multivariate model to adjust the confounding variables. Receiver–operating–characteristic curves (ROC) were used to express the ability of mNUTRIC score for prediction different outcomes via area under curve (AUC). Appropriate cut-offs were identified by highest combined sensitivity (SN) and specificity (SP) using Youden’s index. Positive predictive value (PPV), negative predictive value (NPV), positive likelihood ratio (LR+) and negative likelihood ratio (LR-) to predict outcomes were calculated for mNUTRIC score. According to general guide for the discriminative power of a test based on ROC, AUC between (0.9–1.0), (0.8–0.9), (0.7–0.8), and (0.6–0.7) was considered as excellent, good, fair, and poor, respectively. Statistical analysis was carried out using SPSS software (ver.21) (SPSS Inc. IL, Chicago, USA) and MedCalc (https://www.medcalc.org/calc/diagnostic_test.php). In all analyses, P-values less than 0.05 were considered as significant.

## Results

### Patients’ characteristics

A total of 528 patients were consecutively enrolled in the study over the 24 months period. Eighty-three were excluded due to discharge or death before the second admission day and missing data. Therefore, 445 patients were included in the study. The mean age of the patients was 61.35 ± 10.88 years (median: 64, minimum: 28, maximum: 85 years), with 51.7% (n = 230) male. The most three common reasons of admission were trauma (n = 116, 26.1%), cancer (n = 63, 14.2%), and cerebrovascular accident [CVA] (n = 46, 10.3%). As for comorbidities, hypertension [HTN] (n = 95, 21.3%) and diabetes mellitus [DM] (n = 72, 16.2%) were the most two frequent ones. Emergency department (n = 172, 39%) and surgery room (n = 163, 37%) were the most two frequent type of admission. The mean ± SD of APACHE II and SOFA scores of the patients were 21.07 ± 4.27 and 10.45 ± 2.064, respectively. Blood culture showed 13.9% and 86.1% negative and positive in the patients, respectively. Baseline demographic and clinical characteristics of the patients are shown in Table 1.

**Table 1:** Demographic and clinical characteristics data of all patients (n = 445)

Characteristics		Frequency
Age (years)	Mean ± SD (range)	61.35 ± 10.88 (28–85)
	Median (IQR)	64 (54–70)
Gender	Male/Female (%)	230/215 (51.7)
Comorbidities	Yes (%)	257 (57.8)
Type of comorbidities	CHF (%)	36 (8.1)
	DM (%)	72 (16.2)
	HTN (%)	95 (21.3)
	IHD (%)	62 (13.9)
	HLP (%)	2 (0.4)
	Others	8 (1.8)
Reasons of admission	Embolism (%)	26 (5.8)
	ARDS (%)	16 (3.6)
	CVA (%)	46 (10.3)
	EDH (%)	15 (3.4)
	Encephalopathy (%)	15 (3.4)
	ICH (%)	35 (7.9)
	Cancer (%)	63 (14.2)
	MI (%)	20 (4.5)
	Trauma (%)	116 (26.1)
	Pneumonia (%)	27 (6.1)
	SAH (%)	10 (2.2)
	Sepsis (%)	27 (6.1)
	Brain Tumor (%)	15 (3.4)
	Other (%)	14 (3.1)
Type of admission	Surgery room (%)	163 (37)
(n = 441)	Emergency (%)	172 (39)
	Department (%)	88 (20)
	Elective (%)	18 (4)
APACHE II	Mean ± SD (range)	21.07 ± 4.27 (13–35)
	Median (IQR)	20 (18–23)
SOFA	Mean ± SD (range)	10.45 ± 2.064
	Median (IQR)	10 (9–11)
Blood culture	Positive (%)	62 (13.9)

Abbreviations; Congestive heart failure (CHF), Diabetes mellitus (DM), Hypertension (HTN), Ischemic heart disease (IHD), Hyperkeratosis lenticularis perstans (HLP), Acute respiratory distress syndrome (ARDS), Cerebrovascular accident (CVA), Epidural hematoma (EDH), Intracerebral brain hemorrhage (ICH), Myocardial infarction (MI), Subarachnoid hemorrhage (SAH).

### Nutrition characteristics and outcomes

Based on mNUTRIC score, 62 (13.9%) and 383 (86.1%) individuals were identified at high and low nutritional risk, respectively. Comparison of variables (demographic characteristics, severity of illness, nutrition data and outcomes) according to high (> 4) and low (≤ 4) mNUTRIC score are presented in Table 2. Enteral nutrition was used for 86.5% of all patients, parenteral nutrition for 11.7%, and enteral with parenteral nutrition in 1.8%. Our results showed that the provided amounts of calories and proteins were significantly lower in patients with mNUTRIC score > 4 and also lower than optimal values (< 80%), which were associated with underfeeding [24]. The overall mortality rate, having MV and using vasopressor were (n = 38, 8.5%), (n = 229, 51.5%) and (n = 98, 22%), respectively. Significant differences were observed between high (> 4) and low (≤ 4) mNUTRIC score patients in ICU stay (10.85 ± 4.23 vs. 6.81 ± 3.121, P < 0.001), hospital stay (14.03 ± 4.62 vs. 10.58 ± 3.84, P < 0.001), underwent MV (85.5% vs. 46%, P < 0.001), MV duration (8.55 ± 5.35 vs. 2.36 ± 3.07, P < 0.001), using vasopressor (77.4% vs. 13.1%, P < 0.001), duration of using vasopressor (3.34 ± 2.402 vs. 0.33 ± 0.955, P < 0.001) and mortality rate (61.3% vs. 0). According to the results, poor outcomes were observed in patients with a high mNUTRIC score compared to patients with a low mNUTRIC score.

**Table 2:** Comparison of variables according to high (> 4) and low (≤ 4) mNUTRIC score

Variables		Total patients (n = 445)	Patients with low mNUTRIC score (n = 383)	Patients with high mNUTRIC score (n = 62)	P-value
Age	Mean ± SD	61.35 ± 10.88	61.41 ± 11.08	61.02 ± 9.62	0.791
Gender	Male (%)	230 (51.7)	192 (50.1)	38 (61.3)	0.103
	Female (%)	215 (48.3)	191 (49.9)	24 (38.7)	
Comorbidities	Yes (%)	257 (57.8)	218 (56.9)	39 (62.9)	0.376
	No (%)	188 (42.2)	165 (43.1)	23 (37.1)	
APACHE II	Mean ± SD	21.07 ± 4.27	19.98 ± 2.59	27.75 ± 6.17	< 0.001*
	Median (IQR)	20 (18–23)	20 (18–22)	29 (23–33)	
SOFA	Mean ± SD	10.45 ± 2.06	9.95 ± 1.305	13.58 ± 2.97	< 0.001*
	Median (IQR)	10 (9–11)	10 (9–11)	14.5 (11–16)	
GVR more than	Yes (%)	62 (13.9)	28 (7.3)	34 (54.8)	< 0.001*
200 cc	No (%)	383 (86.1)	355 (92.7)	28 (45.2)	
Dysglycemia	Yes (%)	57 (12.8)	36 (9.4)	21 (33.9)	< 0.001*
	No (%)	388 (87.2)	347 (90.6)	41 (66.1)	
Type of nutrition	Enteral (%)	385 (86.5)	325 (91.9)	33 (53.2)	< 0.001*
	Parenteral (%)	52 (11.7)	31 (8.1)	21 (33.9)	
	Enteral and Parenteral	8 (1.8)	0	8 (12.9)	
Received energy	Mean ± SD	1636.5 ± 136.97	1643.94 ± 128.05	1591.13 ± 177.30	0.005*
(Kcal)	Median (IQR)	1650 (1600–1700)	1650 (1600–1700)	1600 (1500–1700)	
Percentage of	Mean ± SD	83.11 ± 6.54	84.16 ± 5.08	76.61 ± 9.99	< 0.001*
received energy	Median (IQR)	85 (80–85)	85 (80–85)	75 (70–85)	
Received Protein	Mean ± SD	0.803 ± 0.061	0.811 ± 0.051	0.751 ± 0.086	< 0.001*
(gr)	Median (IQR)	0.8 (0.8–0.85)	0.8 (0.8–0.85)	0.75 (0.7–0.85)	
Percentage of	Mean ± SD	87.57 ± 7.49	89.08 ± 5.71	78.22 ± 10.04	< 0.001*
received Protein	Median (IQR)	90 (85–90)	90 (85–90)	75 (70–85)	
MV	Yes (%)	229 (51.5)	176 (46)	53 (85.5)	< 0.001*
	No (%)	216 (48.5)	207 (54)	9 (14.5)	
MV duration	Mean ± SD	6.26 ± 3.72	2.36 ± 3.07	8.55 ± 5.35	< 0.001*
(n = 229)	Median (IQR)	6 (3–8)	6 (3–8)	9 (4–12)	
ICU LOS	Mean ± SD	7.38 ± 3.57 (3–21)	6.81 ± 3.121	10.85 ± 4.23	< 0.001*
	Median (IQR)	6 (4–10)	6 (4–10)	11 (8–14)	
Hospital LOS	Mean ± SD	11.06 ± 4.13	10.58 ± 3.84	14.03 ± 4.62	< 0.001*
	Median (IQR)	10 (7–14)	10 (7–14)	14.5 (10–18)	
Vasopressor	Yes (%)	98 (22)	50 (13.1)	48 (77.4)	< 0.001*
	No (%)	347 (78)	333 (89.6)	14 (22.6)	
Days of	Mean ± SD	3.39 ± 1.78	0.33 ± 0.955	3.34 ± 2.402	< 0.001*
Vasopressor (n = 98)	Median (IQR)	3 (2–4)	3 (2–4)	4 (1–4)	
Mortality	Yes (%)	38 (8.5)	0	38 (61.3)	< 0.001*
	No (%)	407 (91.5)	383 (100)	24 (38.7)	

Abbreviations; Gastric residual volume (GRV), Mechanical ventilation (MV), Length of stay (LOS)

### Logistic regression findings

Univariable and multivariable binary logistic regression analysis to evaluate associations of various baseline parameters and mNUTRIC score to predict ICU LOS, hospital LOS and underwent MV are presented in Table 3. Univariable logistic regression modeling demonstrated that the having comorbidities, higher scores of SOFA and APACHE II, positive blood culture, high mNUTRIC score (> 4), GVR > 200 cc, and having dysglycemia, were associated with longer ICU-LOS, hospital-LOS and underwent MV. In multivariable analysis, the risk of longer ICU-LOS was increased with age over 64 years old (OR: 2.312, 95%CI: 1.375–3.887, P = 0.002), higher SOFA score (OR: 2.077, 95%CI: 1.334–3.236, P = 0.001), positive blood culture (OR: 2.692, 95%CI: 1.145–6.329, P = 0.023), GVR > 200 cc (OR: 2.857, 95%CI: 1.193–6.838, P = 0.018), and dysglycemia (OR: 2.327, 95%CI: 1.298–4.910, P < 0.001). Multivariable analysis showed that the comorbidity (OR: 1.696, 95% CI: 1.057–2.722, P = 0.029), higher SOFA score (OR: 1.823, 95%CI: 1.196–2.780, P = 0.005), GVR > 200 cc (OR: 3.788, 95% CI: 1.235–11.621, P *<* 0.020) and dysglycemia (OR: 1.311, 95% CI: 1.196–3.494, P < 0.001), were associated with longer hospital-LOS. In addition, multivariable analysis demonstrated that the comorbidity (OR: 1.883, 95%CI: 1.129–3.140, P = 0.015), higher score of SOFA (OR: 2.466, 95%CI: 1.555–3.911, P < 0.001), positive blood culture (OR: 4.036, 95%CI: 1.601–5.175, P = 0.003), GVR > 200 (OR: 3.449, 95%CI: 1.423–8.361, P = 0.006) and dysglycemia (OR: 3.595, 95%CI: 1.968–5.918, P = 0.001) could be increased the risk of MV.

**Table 3:** Univariable and multivariable binary logistic regression analysis to evaluate associations of various baseline parameters and mNUTRIC score to predict MV duration, ICU and hospital stay

Variables	Univariate		Multivariate	
	**OR (95% CI)**	***P*** **-value**	**OR (95% CI)**	***P*** **-value**
ICU length of stay				
Age (> 64 vs. ≤64 years)	1.008 (0.991–1.025)	0.368	2.312 (1.375–3.887)	0.002*
Gender (Male vs. Female)	1.048 (0.722–1.52)	0.806	0.882 (0.549–1.415)	0.603
Comorbidity (Yes vs. No)	1.951 (1.331–2.858)	0.001*	1.487 (0.897–2.465)	0.124
SOFA	2.161 (1.817–2.572)	0.001*	2.077 (1.334–3.236)	0.001*
APACHE II	1.414 (1.301–1.535)	< 0.001*	1.008 (0.810–1.255)	0.941
Blood culture (Positive vs. Negative)	5.221 (2.694–10.116)	< 0.001*	2.692 (1.145–6.329)	0.023*
mNUTRIC score (> 4 vs. ≤4)	6.655 (3.284–13.485)	< 0.001*	1.105 (0.370–3.302)	0.859
GVR > 200 CC (Yes vs. No)	3.803 (2.056–7.036)	< 0.001*	2.857 (1.193–6.838)	0.018*
Dysglycemia (Yes vs. No)	3.167 (8.930–4.550)	< 0.001*	2.327 (1.298–4.910)	< 0.001*
Hospital length of stay				
Age (> 64 vs. ≤64 years)	1.006 (0.989–1.024)	0.460	1.527 (0.952–2.450)	0.079
Gender (Male vs. Female)	0.958 (0.66–1.39)	0.821	0.808 (0.521–1.255)	0.343
Comorbidity (Yes vs. No)	1.994 (1.36–2.924)	< 0.001*	1.696 (1.057–2.722)	0.029*
SOFA	1.715 (1.487–1.977)	< 0.001*	1.823 (1.196–2.780)	0.005*
APACHE II	1.270 (1.187–1.359)	< 0.001*	0.953 (0.774–1.173)	0.647
Blood culture (Positive vs. Negative)	3.844 (2.078–7.111)	< 0.001*	1.667 (0.780–3.564)	0.187
mNUTRIC score (> 4 vs. ≤4)	3.844 (2.078–7.111)	< 0.001*	1.191 (0.861–2.398)	0.202
GVR > 200 CC (Yes vs. No)	3.490 (1.909–6.381)	< 0.001*	2.901 (1.299–6.481)	0.009*
Dysglycemia (Yes vs. No)	3.720 (1.288–4.933)	< 0.001*	3.935 (1.316–5.767)	0.001*
MV				
Age (> 64 vs. ≤64 years)	0.998 (0.981–1.015)	0.775	1.022 (0.613–1.703)	0.934
Gender (Male vs. Female)	0.952 (0.656–1.381)	0.796	0.760 (0.472–1.223)	0.258
Comorbidity (Yes vs. No)	1.863 (1.273–2.725)	0.001*	1.883 (1.129–3.140)	0.015*
SOFA	2.221 (1.859–2.654)	< 0.001*	2.466 (1.555–3.911)	< 0.001*
APACHE II	1.407 (1.295–1.528)	< 0.001*	0.910 (0.726–1.140)	0.410
Blood culture (Positive vs. Negative)	8.023 (3.717–17.314)	< 0.001*	4.036 (1.601–5.175)	0.003*
mNUTRIC score (> 4 vs. ≤4)	6.92 (3.322–14.439)	< 0.001*	1.065 (0.347–3.264)	0.913
GVR > 200 CC (Yes vs. No)	4.251 (2.233–8.09)	< 0.001*	3.449 (1.423–8.361)	0.006*
Dysglycemia (Yes vs. No)	69.59 (9.537–90.85)	< 0.001*	3.595 (1.968–5.918)	0.001*

*P < 0.05 considered as significant, Abbreviations: Odds ratio (OR), Confidence Interval (CI)

Univariable and multivariable binary logistic regression analysis to evaluate associations of various baseline parameters and mNUTRIC score to predict ICU mortality and duration of MV and vasopressor are presented in Table 4. Univariable logistic regression modeling demonstrated that the having comorbidities, higher scores of SOFA and APACHE II, positive blood culture, mNUTRIC score (> 4), GVR > 200 cc and having dysglycemia were associated with higher ICU mortality rate, prolonged MV duration and longer duration of using vasopressor. In multivariable analysis, the risk of ICU mortality was increased with having comorbidities (OR: 2.289, 95%CI: 1.006–5.211, P = 0.048), positive blood culture (OR: 4.140, 95%CI: 1.806–5.492, P = 0.001), mNUTRIC score > 4 (OR: 6.970, 95%CI: 1.372–8.423, P = 0.019) and GVR > 200 cc (OR: 4.317, 95%CI: 1.955–5.533, P = 0.001). Based on multivariable analysis, age over 64 years (OR: 1.934, 95%CI: 1.017–3.678, P = 0.044), higher SOFA score (OR: 2.823, 95%CI: 1.553–5.130, P = 0.001) and dysglycemia (OR: 4.069, 95%CI: 3.668–5.420, P = 0.001) were associated with prolonged MV duration. Moreover, multivariable model showed that the age over 64 years (OR: 2.724, 955CI: 1.043–4.113, P = 0.041), mNUTRIC score (> 4) (OR: 5.898, 95%CI: 2.720–6.024, P = 0.001) and dysglycemia (OR: 4.116, 95%CI: 2.803–6.069, P = 0.001) could be increased the risk of longer using vasopressor.

**Table 4:** Univariable and multivariable binary logistic regression analysis to evaluate associations of various baseline parameters and mNUTRIC score to predict mortality and duration of MV and vasopressor

Variables	Univariate		Multivariate	
	**OR (95% CI)**	***P*** **-value**	**OR (95% CI)**	***P*** **-value**
ICU Mortality				
Age	1.009 (0.978–1.041)	0.570	1.238 (0.635–2.412)	0.531
Gender (Male vs. Female)	1.673 (0.841–3.325)	0.142	1.669 (0.839–3.319)	0.144
Comorbidity (Yes vs. No)	2.530 (1.168–5.480)	0.019*	2.289 (1.006–5.211)	0.048*
SOFA	7.205 (3.684–14.091)	< 0.001*	2.518 (0.404–5.677)	0.322
APACHE II	2.570 (1.826–3.616)	< 0.001*	1.744 (0.629–4.837)	0.285
Blood culture (Positive vs. Negative)	6.512 (3.200-13.25)	< 0.001*	4.140 (1.806–5.492)	0.001*
mNUTRIC score (> 4 vs. ≤4)	7.775 (2.097–11.957)	< 0.001*	6.970 (1.372–8.423)	0.019*
GVR > 200 CC (Yes vs. No)	5.708 (2.797–11.648)	< 0.001*	4.317 (1.955–5.533)	0.001*
Dysglycemia (Yes vs. No)	4.938 (2.377–6.257)	< 0.001*	1.836 (0.742–4.543)	0.189
MV duration				
Age	1.000 (0.981–1.019)	0.982	1.934 (1.017–3.678)	0.044*
Gender (Male vs. Female)	1.213 (0.802–1.833)	0.360	0.970 (0.533–1.763)	0.920
Comorbidity (Yes vs. No)	1.637 (1.066–2.514)	0.024*	1.123 (0.583–2.165)	0.728
SOFA	3.358 (2.581–4.370)	< 0.001*	2.823 (1.553–5.130)	0.001*
APACHE II	1.691 (1.510–1.895)	< 0.001*	1.072 (0.808–1.423)	0.629
Blood culture (Positive vs. Negative)	2.990 (1.727–5.176)	< 0.001*	0.659 (0.268–1.624)	0.365
mNUTRIC score (> 4 vs. ≤4)	3.004 (2.265–3.984)	< 0.001*	1.118 (0.341–3.661)	0.854
GVR > 200 CC (Yes vs. No)	3.499 (2.018–6.067)	< 0.001*	1.683 (0.633–4.474)	0.296
Dysglycemia (Yes vs. No)	2.437 (1.399–4.175)	< 0.001*	4.069 (3.668–5.420)	0.001*
Duration of vasopressor				
Age	1.011 (0.986–1.036)	0.380	2.724 (1.043–4.113)	0.041*
Gender (Male vs. Female)	1.698 (0.995–2.898)	0.052	1.555 (0.650–3.718)	0.321
Comorbidity (Yes vs. No)	1.880 (1.073–3.295)	0.027*	1.056 (0.396–2.817)	0.913
SOFA	2.940 (2.259–3.827)	< 0.001*	2.077 (0.879–4.908)	0.096
APACHE II	1.641 (1.456–1.850)	< 0.001*	1.065 (0.698–1.626)	0.770
Blood culture (Positive vs. Negative)	6.024 (3.308–8.972)	< 0.001*	2.423 (0.849–6.911)	0.098
mNUTRIC score (> 4 vs. ≤4)	5.626 (3.765–8.409)	< 0.001*	5.898 (2.720–6.024)	0.001*
GVR > 200 CC (Yes vs. No)	4.994 (2.737–9.113)	< 0.001*	0.735 (0.200-2.706)	0.644
Dysglycemia (Yes vs. No)	5.541 (5.667–6.605)	< 0.001*	4.116 (2.803–6.069)	0.001*

*P < 0.05 considered as significant, Abbreviations: Odds ratio (OR), Confidence Interval (CI)

### Predicting outcomes by mNUTRIC score

**Fig. 1:**
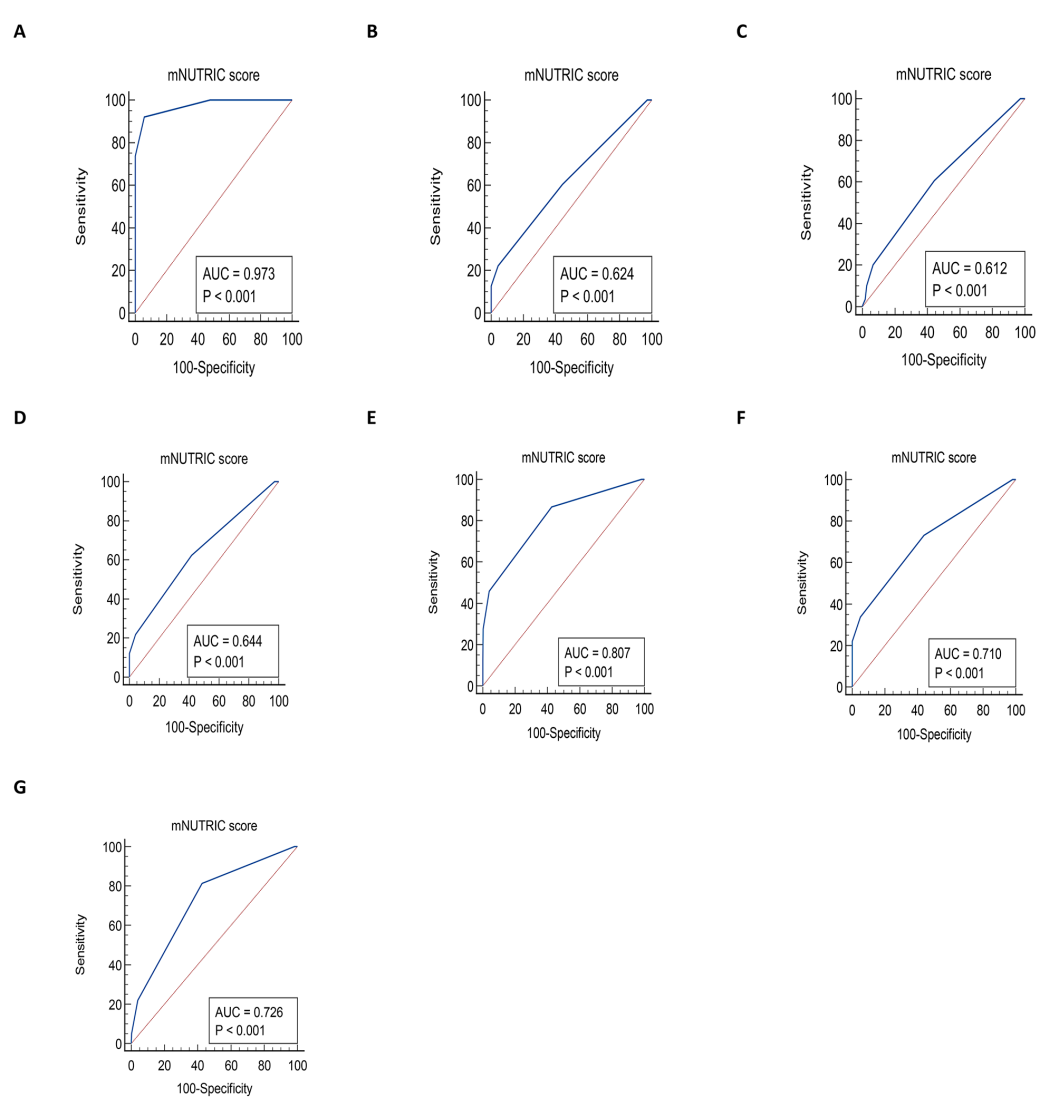
ROC curves of mNUTRIC sore to predict (A) mortality, (B) ICU-LOS > 6 days, (C) hospital-LOS > 10 days, (D) mechanical ventilator, (E) vasopressor, (F) duration of MV ≥ 6 days, and (G) duration of vasopressor ≥ 3 days

Table 5 shows mNUTRIC score performance to predict outcomes with cutoff points. An excellent predictive performance of the mNUTRIC score was found regarding ICU mortality and the area under the ROC curves was (AUC: 0.973, 95% CI: 0.954–0.986, P < 0.001), and the best cutoff value (> 4) had a value sensitivity 92.11%, specificity 94.1%, PPV 59.2%, NPV 99.2%, (LR+) 15.62, (LR-) 0.084, and 0.86% of Yuden index. A good predictive performance of the mNUTRIC score was found regarding using vasopressor and the area under the ROC curves was (AUC: 0.807, 95% CI: 0.767–0.843, P < 0.001), and the best cutoff value (> 3) had a value sensitivity 86.73%, specificity 57.35%, PPV 36.5%, NPV 93.9%, (LR+) 2.03, (LR-) 0.23, and 0.44% of Yuden index. Fair predictive performance of the mNUTRIC score was found regarding duration of using vasopressor (AUC: 0.726, 95% CI: 0.680–0.769, P < 0.001) and also MV duration (AUC: 0.710, 95% CI: 0.666–0.752, P < 0.001) with the best cutoff value at > 3 and 0.38% and 0.29% of Yuden index, respectively. Poor predictive performance of the mNUTRIC score was found regarding ICU LOS (AUC: 0.624, 95% CI: 0.577–0.669, P < 0.001), hospital LOS (AUC: 0.612, 95% CI: 0.565–0.658, P < 0.001), and MV (AUC: 0.644, 95% CI: 0.598–0.689, P < 0.001). The ROC curves for mNUTRIC score performance to predict outcomes are presented in Fig. 1.

**Table 5:** Roc curve results of mNUTRIC score to predicting outcomes Abbreviations; LOS: Length of stay, MV: Mechanical ventilation, CI: Confidence interval, SN: Sensitivity; SP: Specificity; LR+: Positive Likelihood Ratio; LR-: Negative Likelihood Ratio; PPV: Positive Predictive value; NPV: Negative Predictive value, *P < 0.05 considered as significant

Outcomes	AUC (95% CI)	P-value	SN (95% CI)	SP (95% CI)	PPV (95% CI)	NPV (95% CI)	LR+ (95% CI)	LR- (95% CI)	Youden Index	Cut-point
ICU LOS (> 6 vs. ≤6 days)	0.624 (0.577–0.669)	< 0.001*	22.27 (17.0-28.4)	95.56 (92.0-97.8)	83.0 (71.8–90.4)	55.7 (53.9–57.6)	5.01 (2.61–9.64)	0.81 (0.75–0.88)	0.178	> 4
Hospital LOS (> 10 vs. ≤10 days)	0.612 (0.565–0.658)	< 0.001*	60.73 (53.9–67.2)	55.75 (49.0-62.3)	57.0 (52.5–61.3)	59.5 (54.6–64.3)	1.37 (1.15–1.64)	0.70 (0.58–0.86)	0.164	> 3
MV (Yes vs. No)	0.644 (0.598–0.689)	< 0.001*	62.45 (55.8–68.7)	58.33 (51.5–65.0)	61.4 (56.9–65.7)	59.4 (54.5–64.1)	1.50 (1.24–1.81)	0.64 (0.53–0.79)	0.207	> 3
MV duration (≥ 6 vs. <6 days)	0.710 (0.666–0.752)	< 0.001*	73.23 (64.6–80.7)	55.97 (50.3–61.5)	39.9 (36.0-43.8)	84.0 (79.5–87.7)	1.66 (1.41–1.96)	0.48 (0.35–0.65)	0.292	> 3
Vasopressor (Yes vs. No)	0.807 (0.767–0.843)	< 0.001*	86.73 (78.4–92.7)	57.35 (52.0-62.6)	36.5 (33.2–39.9)	93.9 (90.2–96.2)	2.03 (1.76–2.35)	0.23 (0.14–0.39)	0.440	> 3
Duration of vasopressor (≥ 3 vs. <3 days)	0.726 (0.680–0.769)	< 0.001*	81.36 (69.1–90.3)	57.35 (52.0-62.6)	25.3 (22.2–28.7)	94.5 (91.0-96.7)	1.91 (1.61–2.27)	0.33 (0.19–0.56)	0.387	> 3
Mortality (Yes vs. No)	0.973 (0.954–0.986)	< 0.001*	92.11 (78.6–98.3)	94.10 (91.4–96.2)	59.2 (49.3–68.4)	99.2 (97.7–99.7)	15.62 (10.4–23.2)	0.084 (0.028–0.25)	0.862	> 4

## Discussion

This study was an analysis of a prospective observational study at general ICUs. We used mNUTRIC score in an attempt to demonstrate an association between nutritional risk and mortality in critically ill patients. We found almost a high incidence of nutritional risk in ICU patients, which was associated with a poor outcome. Our major findings in the study are as follows. Firstly, 13.9% of critically ill patients admitted to the ICU were categorized as being at high nutrition risk (a mNUTRIC score of > 4). Secondly, the group of high nutrition risk was characterized by lower amounts of calories and proteins intake, prolong MV duration, using of vasopressor more than 3 days, longer hospital and ICU stay, and higher rates of mortality. Thirdly, an excellent and good predictive performance of the mNUTRIC score was found regarding ICU mortality and using vasopressor, respectively. However, this predictive was fair for MV and vasopressor duration and poor for ICU and hospital length of stay.

The nutrition assessment in ICU patients presents a special challenge to intensivists [25]. Most of nutrition screening tools before mNUTRIC score were not suitable for critically ill patients because nutritional risk in ICU is linked with inflammation and hypermetabolic state, and the previous tools didn’t include these important causes for nutritional risk [10, 26]. In this regard, the mNUTRIC score is a novel instrument specific for critically ill patients [13]. Many studies have been demonstrated the importance of the mNUTRIC score in prediction of outcomes in critically ill patients [12, 13, 16, 18, 27–29]. Our results are consistent with previous studies indicated that mNUTRIC score was associated with the prolonged-MV, higher ICU-LOS and longer using of vasopressors [12, 16, 28, 30]. Poor nutritional status patients have a poor prognosis, but those with a good nutritional status do not always have better outcomes because of the many other factors associated with their illnesses such as severity of disease and inflammation [18, 27, 28]. The ICU mortality rate in our study was 8.5%, which was quite lower than the previous studies. The range of ICU mortality rate was varying between 10% and 50% depending on the severity of the disease and the population studied [18, 27, 29]. This difference may be because endpoint in our study was ICU mortality however majority of previous studies the endpoint was 28-day mortality after ICU discharge [18, 27, 28, 30]. In this study, we found that the mNUTRIC score was a good prognostic predictor in critically ill patients and that high mNUTRIC scores were associated with an elevated risk of ICU mortality (OR: 6.970, 95%CI: 1.372–8.423, P = 0.019). This finding is consistent with those of prior studies [28, 31–33].

Actually, there is not any death in the group with low mNUTRIC score. The sample size in this group is almost large which can decrease the possibility of sampling error. The patients with low mNUTRIC score had significantly lower energy intake and higher level of dysglycemic events (hyperglycemia, hypoglycemia and glucose variation) which all are independent risk factors for mortality. Although the patients in this group had a less duration of MV and ICU LOS which can result in lower incidence of major life-threatening complications. Our results demonstrated that mNUTRIC score had an excellent performance of predicting ICU mortality (AUC: 0.973), which represents better power than predicting 28-day deaths [12, 30, 31, 33]. Most previous studies have shown fair performance in predicting 28-day mortality with mNUTRIC score in different nationality for example in Brazil, Caucasian and Asian, Dutch and South Korea population AUC to predict 28-mortality were 0.718, 0,710, 0.768 and 0.757, respectively. In line with our results, a recently study by Majari et al. [34], showed that mNUTRIC score had a fair performance of predicting 28-day mortality (AUC: 0.806) in Iranian population. We found that the best cut-off value for the mNUTRIC score to predict mortality was > 4 (sensitivity 92.11% and specificity 94.10%), and the Youden index was 0.862, which is consistent with previous work by de Vries et al. [16], and wang et al. [31]. However, in another study, the best cut-off value was at 6 (sensitivity 75% and specificity 65%), and the Youden index was 0.401 [33]. Jung et al. [35], reported that patients were considered to be at high risk of nutritional risk when their mNUTRIC score was ≥ 5. Our study included patients with various diseases, while Jung’s study population was limited to patients with sepsis. mNUTRIC score is a new predictor of mortality in critically ill patients. It has been shown to be more accurate than existing predictors such as APACHE, SAPS, and SOFA.In a study by Hai et al. (2020) [36], NUTRIC score was compared to APACHE II, SAPS, and SOFA scores in n Patients with Sepsis. The results showed that mNUTRIC score had higher accuracy than APACHE II, SAPS 2, and SOFA scores in predicting mortality, with an area under the receiver operating characteristic curve of 0.79 (sensitivity 67.1% and specificity 81.0%, P < 0.001), 0.78 (sensitivity 84.9% and specificity 67.7%), 0.73(sensitivity 66.1%, specificity 77.7%), and 0.77(sensitivity 76.7% and specificity 65.3%), respectively. Additionally, the calibration of This suggests that NUTRIC score is a better predictor of mortality than existing predictors.

Early identification of patients at high nutritional risk using effective tools is essential to promptly initiate a comprehensive nutritional assessment and appropriate treatment, as well as subsequent improvement in patient outcomes. Based on our findings, the mNUTRIC score is a promising screening tool for nutritional risk in ICU patients. In addition, the validity of the mNUTRIC score was compared with Nutritional Risk Screening (NRS)-2002 Score and Malnutrition Universal Screening Tool (MUST) in Iranian population by Majari et al. [34], showed the superiority of the mNUTRIC score over NRS-2002 and the MUST score in the screening of nutritional risk in ICU patients. All components of this tool can be accessed from patient records without the need for patient or family reviews, enhancing the clinical application of this tool. Most of previous studies showed that a cutoff point of more than 5.5 or 6 for prediction of mortality in critically ill patients. We showed a score of more than 4 as a risk of death. The mentioned point could be due to using mNUTRIC score instead of NUTRIC score as previous studies used the NUTRIC score as a score for mortality prediction in critically ill patients. Also, this could be due to patient selection as we excluded patients who diagnosed as brain dead at admission or during ICU stay, transferred from another ICU or hospital, those readmitted to the ICU and patients who discharged or died within 24 h after ICU admission.

The strengths of this study were the use of prospective design, which adjusted the confounding variables, as well as sampling from the general ICU of two hospitals with heterogeneous patients, allowing the results to be generalized. The limitations of our study were primarily related to the small sample size, which included a limited number of patients in the groups with the highest and lowest mNUTRIC values. Due to the lack of data on indicators of inflammation such as IL6, it was not possible to calculate the NUTRIC score to see the difference between the NUTRIC score and the mNUTRIC score.

In summary, based on mNUTRIC score, 62 (13.9%) of critically ill patients admitted to the ICU were identified at high nutritional risk. The group of high nutrition risk was characterized by lower amounts of calories and proteins intake, prolong MV duration, using of vasopressor more than 3 days, a longer hospital and ICU stay, and higher rates of mortality. According to our findings, an excellent and good predictive performance of the mNUTRIC score was found regarding ICU mortality and using vasopressor, respectively. However, this predictive was fair for MV and vasopressor duration and poor for ICU and hospital stay. In conclusion, the mNUTRIC score is a practical, easy-to-use way based on data which are easy to obtain in the critical care setting.

## Data Availability

The data that support the findings of this study are available from the corresponding author upon reasonable request.

## Abbreviation

mNUTRIC score: Modified Nutrition Risk in the Critically Ill (NUTRIC) score
ICUs: Intensive care units
(ROC)-curve: Operating characteristic
AUC: Area under the curve
MV: Mechanical ventilator
APACHE-II: Acute Physiology and Chronic Health Evaluation II
SOFA: Sequential Organ Failure Assessment
SD: Standard deviation
CVA: Cerebrovascular accident
HTN: hypertension
DM: Diabetes mellitus
MUST: Malnutrition Universal Screening Tool
